# Knock Down of Heat Shock Protein 27 (HspB1) Induces Degradation of Several Putative Client Proteins

**DOI:** 10.1371/journal.pone.0029719

**Published:** 2012-01-04

**Authors:** Benjamin Gibert, Bénédicte Eckel, Lydie Fasquelle, Maryline Moulin, Frantz Bouhallier, Vincent Gonin, Gregory Mellier, Stéphanie Simon, Carole Kretz-Remy, André-Patrick Arrigo, Chantal Diaz-Latoud

**Affiliations:** 1 Centre de Génétique Moléculaire et Cellulaire, CNRS UMR5534, Université Lyon 1, Université de Lyon, Villeurbanne, France; 2 Inserm U583, Institut des Neurosciences, Hôpital Saint Eloi, Montpellier, France; Université Montpellier 1, Montpellier, France; 3 Department of Biochemistry, La Trobe University, Victoria, Australia; 4 INSERM U664, Université Lyon 1, Université de Lyon, Villeurbanne, France; 5 Institut de Génomique Fonctionnelle de Lyon, Université Lyon 1, ENS Lyon, CNRS, UMR5242, INRA, UMR1288, Lyon, France; 6 Apoptosis, Cancer, and Development Laboratory, CRCL UMR INSERM U1052-CNRS UMR5286, Université de Lyon, Centre Léon Bérard, Lyon, France; University Medical Center Groningen, University of Groningen, The Netherlands

## Abstract

Hsp27 belongs to the heat shock protein family and displays chaperone properties in stress conditions by holding unfolded polypeptides, hence avoiding their inclination to aggregate. Hsp27 is often referenced as an anti-cancer therapeutic target, but apart from its well-described ability to interfere with different stresses and apoptotic processes, its role in non-stressed conditions is still not well defined. In the present study we report that three polypeptides (histone deacetylase HDAC6, transcription factor STAT2 and procaspase-3) were degraded in human cancerous cells displaying genetically decreased levels of Hsp27. In addition, these proteins interacted with Hsp27 complexes of different native size. Altogether, these findings suggest that HDAC6, STAT2 and procaspase-3 are client proteins of Hsp27. Hence, in non stressed cancerous cells, the structural organization of Hsp27 appears to be a key parameter in the regulation by this chaperone of the level of specific polypeptides through client-chaperone type of interactions.

## Introduction

Hsp27, also called HspB1, is a member of the small heat shock family of proteins that are characterized by their conserved C-terminal α-crystallin domain [1]. This oligomeric phosphoprotein bears an ATP-independent chaperone activity [2]. Hsp27 is also known for its anti-apoptotic activities [3] that appear of complex nature because of the dynamic and specific changes in the structural organization of this protein in response to various stimuli. Hence, multiple strategies appear to be set up by Hsp27 to interfere with apoptotic processes [4]. Indeed, Hsp27 can interact with several components of the apoptotic machinery. It interferes with apoptotic receptor like CD95-Fas/Apo1 by sequestering Daxx, a polypeptide crucial for death signal transduction [5]. Hsp27 also interacts with cytochrome c, once it is released from mitochondria, hence preventing apoptosome formation [6]. A binding to procaspase-3 has been observed that prevents the cleavage into active caspase-3 [7]. Hsp27 also interferes with targets upstream of mitochondria, as for example F-actin, hence preventing its disruption and aggregation. This delays the accumulation of cytochrome *c* in the cytosol and subsequently reduces caspases activation [8]. Hsp27 is phosphorylated by the p38MAPK/MAPKAPK2 pathway and promotes the activation of the pro-survival serine/threonine kinase Akt [9], [10].

Hsp27 is well referenced as a therapeutic target in cancer [11] since its increased expression in several types of tumor cells correlates with increased aggressiveness, lack of response to therapies and bad prognostic for patients [12], [13]. For example, Hsp27 expression enhances the resistance to chemotherapeutic drugs like cisplatin, adriamycin and bortezomib [14], [15] and protects against radio-therapeutic radiations, probably as a consequence of its ability to act as an anti-oxidant polypeptide [16]. Thus, targeting Hsp27 level by antisense strategies sensitizes cells to γ-rays [17].

The molecular chaperone distinctiveness of Hsp27 implicates that this constitutively expressed protein could directly interfere with several target proteins and regulates numerous cellular processes. In this regard, one member of the heat shock protein family, Hsp90, is well characterized to interact with an important number of client proteins implicated in cell cycle regulation, signal transduction or gene transcription [18], [19]. By doing so, Hsp90 promotes the stability and activity of polypeptides by controlling, through its chaperone activity, their folding. These studies have been facilitated by the existence of specific chemical inhibitors, i.e benzoquinone ansamycin geldanamycin and its less toxic derivative 17-allylamino-17-demethoxygeldanamycin (17-AAG), which bind Hsp90 ATPase box and knock out the chaperone activity. This disrupts Hsp90 interaction with client proteins that are subsequently degraded through the ubiquitin-dependent proteasomal pathway [20]. Such a mechanism is well referenced for Hsp90 but less documented for other chaperones. However, reports have already mentioned decreased levels of procaspase-3, STAT3 and eIF4E [7], [21], [22] in cells devoid of Hsp27. Hence, despite Hsp27 has no ATPase box and no chemical inhibitors are yet available, we tested whether this chaperone could also regulate a set of client proteins.

Using shRNA-mediated depletion, co-immunoprecipitation and protein activity assays, we show here that, in unstressed HeLa cells, Hsp27 is associated with three putative client proteins: histone deacetylase 6 (HDAC6), signal transducer and activator of transcription 2 (STAT2) and procaspase-3; three polypeptides that play major roles in cytoskeleton deacetylation, signal transduction and apoptosis.

## Experimental Procedures

### Cell culture and transfections

All cells were purchased from the ATCC Cell Biology Collection and were grown at 37°C in a humidified atmosphere containing 5% CO_2_. HeLa or MCF-7 cells were grown in Dubelcco's modified Eagle's medium (DMEM) supplemented with 10% heat inactivated fetal calf serum. For transient expression, one day before transfection with the appropriate DNA vector, exponentially growing cells were seeded at a density of 1.5×10^6^ cells/78 cm^2^. According to the Lipofectamine™ reagent procedure (Invitrogen, Cergy Pontoise, France) DNA vector was left on cells for 3 h. Thereafter, cells were washed once with PBS before being further incubated in fresh culture medium. Forty-eight hours after transfection, cells were submitted to the different treatments.

### Gel electrophoresis and immunoblotting

After treatment, cells were immediately rinsed twice in ice-cold PBS and scraped off the dish. At this point, aliquots were withdrawn for determination of protein concentration. Thereafter, cells were lysed in boiling SDS buffer (62.5 mM Tris-HCl, pH 6.8; 1% SDS; 0.1 M dithioerythritol; 0.001% bromophenol blue and 10% glycerol). Cell lysates were subjected to SDS-Polyacrylamide Gel Electrophoresis (SDS-PAGE) performed as previously described [23]. The detection of immunoblots was performed with the ECL™ system (Amersham Life Science, Pantin, France). Autoradiographs were recorded on X-Omat LS films (Eastman Kodak Co, Rochester, NY).

### Sizing Chromatography

HeLa or MCF-7 cells used to prepare cytosolic supernatants for sizing chromatography experiments were grown as detailed above. Cells from five 100-mm culture plates were harvested on ice by scraping and spun (1000×g, 5 min, 4°C). They were then washed and lysed in the column equilibration buffer (20 mM Tris, pH 7.4; 5 mM MgCl_2_; 20 mM NaCl; 0.1 mM EDTA) supplemented with 0.1% Triton X-100. Cell lysates were spun (10,000×*g*, 10 min) and supernatants were loaded on a sepharose CL-6B column (Sigma, St Louis, MO). Columns fractions were analyzed by immunoblotting as previously described [6]. Molecular-mass markers used to calibrate the gel-filtration column included carbonic anhydrase (29 kDa), albumin (66 kDa), alcohol dehydrogenase (150 kDa), β-amylase (200 kDa), apoferritin (440 kDa), thyroglobulin (669 kDa) and Dextran Blue (>2000 kDa) (Sigma, St Louis, MO).

### Co-Immunoprecipitation experiments (Co-IP)

Co-IP experiments were performed with samples from the sizing columns (see above) known to contain the targeted polypeptides. 2 ml samples of the pooled column fractions were incubated (1 h, 4°C) with Hsp27 antibody (goat polyclonal anti-Hsp27 antibody, Santa Cruz Biotechnologies-Clinisciences, Montrouge, France) followed by incubation (1 h, 4°C) with protein G sepharose (50 µl of a 50% bead slurry per sample, GE Healthcare, Vélizy, France). Samples were briefly centrifuged (5,000×*g*, 30 s), supernatants were discarded and bound proteins were eluted from beads. Detection of co-immunoprecipitated proteins was performed in immunoblots probed with the corresponding antibodies.

### ShRNA construction

The pSuperNeo plasmid (Oligoengine, Madison, WI) was used for DNA vector-based shRNA construction. Based on the cDNA of Hsp27 (HUGO gene nomenclature committee accession No. HGNC:5246) and the shRNA designing tool provided freely by Ambion at its website (http://www.ambion.com), we synthesized DNA templates encoding Hsp27-specific and control shRNAs. The targeted oligonucleotide sequence was: 5′-GCTGCAAAATCCGATGAG-3′. After annealing, ligation and transformation into competent DH5α bacteria (Invitrogen, IllKirch, France), positive colonies were selected through their antibiotic resistance. The correct sequences of the final DNA preparations were confirmed by sequencing (GenomExpress, Meylan, France). pSuperNeo-MsRNA27 and pSuperNeo-ScRNA27 (scramble, Sc27) were designed respectively as degenerated (Ms) and scramble (Sc) controls from the above mentioned sequence of Sh27. pCI-Neo vector was used as a positive control of cell transfection. ShRNA-HDAC6 and its random controls will be described elsewhere.

### Generation of stable HeLa and MCF-7 cells depleted in Hsp27

Five cell culture dishes were seeded at 1×10^5^ cells/78 cm^2^. Transfection of HeLa and MCF-7 cells was performed as described above. One day later, positively transfected cells were selected by neomycin at a concentration of 0.5 (HeLa) or 1 (MCF-7) mg/ml. The level of Hsp27 in neomycin resistant clones was then tested by immunobloting. Five independent clones of each cell line expressing reduced levels of Hsp27, named: HSh27 (HeLa) and MSh27 (MCF-7) and four independent mismatch control clones expressing normal levels of Hsp27 (named: HMs27 and MMs27) were selected, propagated and analyzed.

### Determination of STAT2 transcriptional activity

The different cell lines were transiently transfected with a STAT1/STAT2-responsive luciferase construct encoding the firefly luciferase reporter gene under the control of a minimal (m)CMV promoter and tandem repeats of the interferon stimulated response element (ISRE) (SABiosciences, Frederick, MD, USA). Cells were treated 6 h with 50 U/mL interferon-α to activate the STAT2 interferon response. In this experiment, Hsp27 phosphorylation was inhibited by SB203580 and SB202190 (p38MAPkinase and MK2 inhibitors, respectively) (Sigma, St Louis, MO).

### Quantitative PCR

To assay Caspase3, HDAC6, STAT2 and 3 expression, 1 µg of TRIzol extracted total RNA was reverse-transcribed using the SuperScriptIII kit (Invitrogen, Cergy Pontoise, France). Real-time quantitative PCR was performed on the MX-3000P cycler (Agilent, Massy, France) using QuantiTect SYBR Green kit and QuantiTect Primer Assays (Qiagen, Courtaboeuf, France). All reactions were run in triplicate and gene expression levels were normalized to the ribosomal gene RPS17 using the ΔΔCt method. The sequences of the primers are available upon request.

### Cell death and/or cell survival determination

Twenty-four hours after transfection, cells were seeded in 96-wells plates (7.5×10^3^/well). Twelve hours later, cells were treated for 18 h with staurosporine (Sigma, St Louis, MO). After treatment, the culture medium was discarded and the remaining viable cells were rinsed twice with PBS buffer and stained for 15 min with 0.5% crystal violet in 50% methanol. Afterward, plates were rinsed and dried. Thereafter, the stained cells were solubilized using 0.1 M sodium citrate (pH 5.4) in 20% methanol. The absorbance of each well was read at 570 nm with a Wallac 1420 Multilabel Counter (PerkinElmer, Courtabœuf, France). The percentage of cell survival was based on the ratio of the relative absorbance of the different samples to that of untreated cells [24], [25].

Cell proliferation was determined using the WST-1 assay, which requires a 4 h incubation of cells with the tetrazolium WST-1 salt (4-[3-(4-Iodophenyl)-2-(4-Bonitrophenyl)-2H-5-tetrazolio]-1,3-benzene disulfonate) (Roche, Basel, Switzerland) (10 µl/well) followed by absorbance measurement at 450 nm. The percentage of cellular proliferation was calculated based on a control absorbance.

Cell cycle analysis was realized as previously described [26]. Briefly, cells were fixed in 70% ethanol, then treated with RNAse A, stained with propidium iodide and analyzed by flow cytometry.

### Immunofluorescence analysis

Cells growing on glass cover slips were fixed for 5 min with freshly prepared 4% para-formaldehyde, pH 7.0. Cells were then permeabilized for 5 min in PBS containing 0.1% Triton ×100. The cover slips were incubated for one hour with monoclonal anti-Hsp27 antibody (Stressgen, San Diego, Ca). After washing, cover slips were further incubated for one hour with FITC-conjugated goat anti mouse immunoglobulin. Actin detection was performed using Alexa Fluor™ 488 Phalloidin (Alexa Fluor, Raleigh, NC). Hoechst staining was used to enumerate nuclei and analyze their morphology. Microphotographs were realized using appropriate filters with Zeiss Axioskop microscope equipped with a 63× lens and a digital camera device.

## Results

### shRNA targeting of Hsp27 stimulates staurosporine-mediated apoptosis of two cancer cell lines

**Figure 1:**
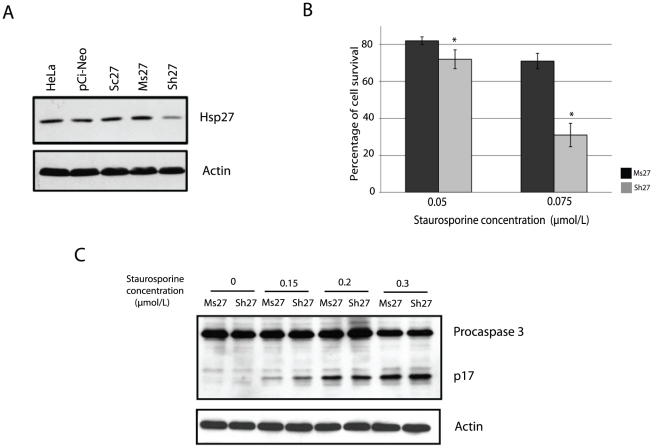
Transient down regulation of Hsp27 protein by shRNA sensitizes HeLa cells to cell death and promotes apoptosis. *A*, pCI-Neo, pSuperNeo-ScRNA27 (Sc27), pSuperNeo-MsRNA27 (Ms27) and pSuper-ShRNA27 (Sh27) vectors were transiently expressed in HeLa cells. Samples were collected 48 h after transfection and Hsp27 level was analyzed by western blot. Level of actin was used as control. *B*, Sensitivity to cell death was determined by crystal violet analysis, as described in Materials and methods. Staurosporine, a pro-apoptotic kinase inhibitor, was used to induce apoptosis (*p*<0.01). *C*, HeLa cells, treated for 4 h with different concentrations of staurosporine, were analyzed by western blot using an anti-caspase3 antibody.

HeLa cells were transiently transfected with pCI-Neo vector (pCI-Neo), pSuperNeo-ScRNA27 (scramble, Sc27), pSuperNeo-MsRNA27 (mismatch, Ms27) and pSuper-ShRNA27 (Sh27). 48 h after transfection, the level of Hsp27 was determined by western blot analysis. As shown in figure 1.A, Sh27 strongly reduced Hsp27 expression whereas the control RNAs did not. Hsp27 is characterized by its strong anti-apoptotic activity, we therefore analyzed whether a decreased level of Hsp27 could sensitize cells to apoptosis. For this, Sh27 depleted cells were treated 18 h with different concentrations of the kinase inhibitor staurosporine. A cell death was increased in Sh27 cells after a 0.075 µmol/L treatment in comparison to control cells (Fig. 1.B). A similar observation was made in transiently transfected MCF-7 cells (data not shown). We also analyzed the activation of caspase-3, characterized by the generation of p12 and p17 fragments from inactive pro-caspase-3 precursor. Immunoblot analysis was performed after 3 h of treatment at different concentrations of staurosporine. A higher level of processed p17 fragment was noticed in Sh27 cells compared to control Ms27 cells. This confirms our preceding observations, using an anti-sense strategy [4], [8], that depletion of Hsp27 stimulates caspase-3 activity and apoptosis of HeLa cells (Figure 1.C).

### Hsp27 constitutive depletion increases the percentage of cells in G2M and modulates the expression of pro- and anti-apoptotic polypeptides

**Figure 2:**
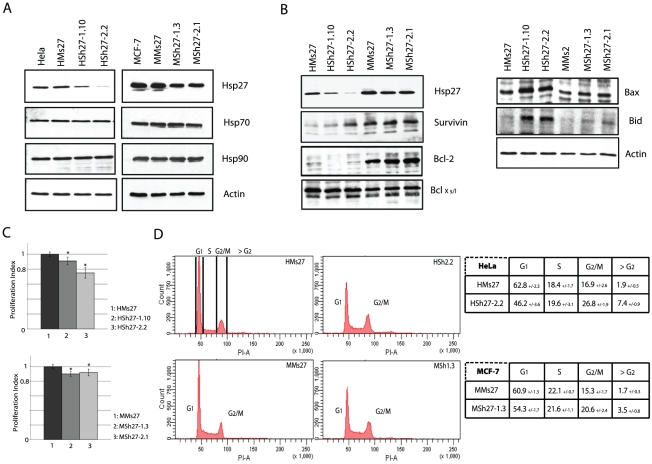
Hsp27 knock down clones, enriched in G2/M cells, show decreased cell proliferation. Constitutive Hsp27 depleted clones of HeLa or MCF-7 were isolated as described in Materials and methods. *A*, HeLa or MCF-7 samples from independent Hsp27 depleted clones were collected and levels of Hsp27, 70 and 90 were analyzed by western blot. *B*, Immunoblot analysis of Hsp27, survivin, Bcl-2, Bcl_Xs/l_, Bax, Bid and actin present in both HeLa and MCF-7 depleted clones. *C*, WST-1 test was performed each 12 h during five days. Proliferation index was determined and reported for each cell line (*p*<0.01). *D*, Cell cycle study was performed by flow cytometry analysis after PI staining as previously described [26]. Quantifications experiments were realized and reported in tables presented on the left of the flow cytometry analysis.

To analyze the consequences of a constitutive depletion of Hsp27, we next produced HeLa and MCF-7 cells stably transfected with the Sh27 vector. Independent clones were isolated and the level of Hsp27 was analyzed (Figure 2.A). HeLa clones HSh27-1.10 and HSh27-2.2 were depleted up to 70% and 95%, respectively; whereas, the level of Hsp27 in MCF-7 clones MSh27-1.3 and MSh27-2.1 was similarly decreased of only about 60%. The lower efficiency observed in MCF-7 cells may result of the ten fold higher level of Hsp27 in these cells compared to HeLa cells (Fig. 2.A) [27]. As it is well referenced that targeting one member of the Hsps family could modify the expression of other Hsps [20], their levels were investigated. As seen in Fig. 2.A, the constitutive depletion of Hsp27 did not induce significant modifications of Hsp70 and Hsp90 levels in either HeLa or MCF-7 cells. Since Hsp27 has been referred as a survival protein [28], the levels of other proteins in this family as well as some members of the apoptotic machinery were also analyzed. As shown in Fig. 1.B, the depletion of Hsp27 induced important changes in the level of several pro- and anti-apoptotic members of the BH3 family. Survivin, an anti-apoptotic BH3 mimetic protein was over-expressed in Hsp27 depleted HeLa cells; its increased level was inversely correlated to the decrease in Hsp27 level. In contrast, the level of survivin was not altered in MCF-7 clones. The reverse phenomenon was detected for Bcl2. This protein had an increased level in Hsp27 partially depleted MCF-7 cells but not in HeLa cells. In both types of cells, the level of Bcl_XS/L_ was not modified. In contrast, the pro-apoptotic proteins Bax and Bid were up regulated in both cell lines (Figure 2.B). Hence, cell adaption to Hsp27 depletion correlates with drastic changes in several anti- and pro- apoptotic polypeptides.

To further characterize the consequences of Hsp27 depletion, a WST-1 test was performed to determine the efficiency of cell proliferation. Intriguingly, the proliferation index was decreased in HeLa and MCF-7 cells lines under-expressing Hsp27. A 10% (HSh27-1.10) and 20% (HSh27-2.2) decrease in the proliferation index of HeLa clones was detected. The two clones of MCF-7 cells showed a 10% decrease (Fig. 2.C).

In order to determine whether the reduced proliferation was linked to cell cycle modifications, exponentially growing cells were propidium iodide stained and analyzed by flow cytometry. In both types of Hsp27 depleted cell lines, the percentage of cells in G2/M phase was increased (Fig. 2.D). 26.8% of HSh27-2.2 cells were in G2/M in contrast to only 16.9% in the control cell line. In depleted MCF-7 clones a similar 5.3% increase in G2/M phase was observed. Furthermore, in Hsp27 depleted cells, we detected an increase in the level of sub-G1 cells. 7.4% were detected in Sh27 cells versus 1.9% in control HeLa cells. A similar phenomenon was observed in MCF-7 cells with an increase from 1.7% to 3.5%. A phenomenon which suggests a slight increase in the ability of cells to commit spontaneous apoptosis.

### Hsp27 is essential to maintain cytoskeleton integrity through an HDAC6 dependent mechanism

**Figure 3:**
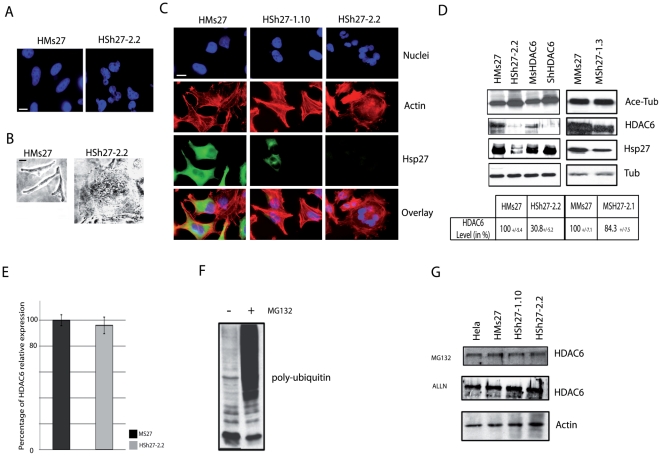
HDAC6 is degraded in Hsp27 deficient cell lines. *A*, photographs of nuclei of Hsp27 depleted HeLa cells stained by Hoechst. *B*, phase contrast pictures of a depleted HeLa clone showing a giant cell containing more than twenty nuclei. Bar = 10 µm. *C*, Fluorescence photomicrographs analysis of Hsp27 and F-actin. HeLa cells were plated on glass cover slips and allowed to enter exponential growing phase for one day. Thereafter, cells were fixed and exposed to Hsp27 mouse monoclonal antibody followed by a goat anti-mouse FITC-conjugated antibody. Cells were also treated with TRITC-labeled phalloidin to visualize F-actin and with Hoechst to detect nuclei. Cells were then examined under a fluorescent microscope and photomicrographs including overlay analysis were recorded. Note the drastic modification of cells morphology induced by Hsp27 withdrawal. Bar = 10 µm. *D*, Transient transfection with ShRNA-HDAC6 and MsRNA-HDAC6 encoding vectors as a control of tubulin induced hyperacetylation. Cell samples were collected and analyzed by immunobloting probed with the indicated antibodies. Quantification of western blot was reported on a table. *E*, quantification of *hdac6* gene relative expression by qPCR analysis. *F*, proteasome activity, blocked by a low dose of MG132 (0.5 µmol/l- 20 hours), inversely correlates with poly-ubiquitin accumulation, as revealed by immunoblot analysis. Cells were submitted to 20 h treatments with either 10 µmol/L ALLN or 0.5 µmol/L MG132, calpain and proteasome inhibitors, respectively. Levels of HDAC6 were revealed by immunobloting.

To better characterize the reduced proliferation efficiency observed in Hsp27 depleted cells, we analyzed the morphology of the different cells lines. HeLa cells were first stained with Hoechst to analyze nucleus morphology. As shown in Fig. 3.A, Hsp27 depleted HeLa cells showed strong nucleus abnormalities. Nuclei were no more spherical and well shaped but morphologically affected. All cells of the highly depleted clone HSh27-2.2 presented nucleus abnormalities and giant cells bearing up to 20 nuclei were frequently observed (Fig. 3.B). This polyploidy confirmed the observation described in Fig. 2.D highlighting the drastic increase in G2 cells. Interestingly, an increase in plurinucleated cells was also detected by flow cytometry analysis in MCF-7 clones, but this increase was not associated with any alterations of nucleus phenotype (data not shown); this is probably due to the high level of Hsp27 which remains in these cells.

Hsp27 is well referenced to support cytoskeletal integrity, particularly by protecting F-actin disruption in stress condition [29], [30]. Consequently, we next performed immunofluorescence analysis of actin cytoskeleton of the different HeLa cell clones. No modification of cytoskeletal integrity was observed (Fig. 3.C). Structure of other filaments like tubulin and vimentin was also not modified (data not shown). Biochemical post-translational modifications of cytoskeleton components were also analyzed. We first analyzed the acetylated form of α-tubulin. This modified tubulin is present in various microtubule structures where it acts as a stabilizer essential in many pathways like cell growth, cell migration and morphogenesis [31]. As revealed by immunobloting, α-tubulin was hyperacetylated in Hsp27 depleted HeLa cells (Fig. 3.D). This observation prompted us to analyze histone deacetylase 6 (HDAC6), the mammalian enzyme responsible of α-tubulin deacetylation [31]. As seen in Fig. 3.D, in Hsp27 depleted HeLa cells, the level of HDAC6 polypeptide was dramatically decreased in a negative correlation with α-tubulin hyperacetylation (Fig. 3.D). Similar increase in α-tubulin hyperacetylation was observed when HDAC-6 level was artificially down regulated with a specific shRNA-HDAC6 (Fig. 3.D). This last phenomenon occurred without altering the level of Hsp27. The same experiments were performed in MCF-7 cells that contain a high level of HDAC6. Only a slight decrease of this protein was observed in MSh27-1.3 cells (Fig. 3.D); a phenomenon that corresponded to the small decrease in Hsp27 level and to no significant increase in α-tubulin acetylation and nucleus aberration phenotype.

To better characterize the mechanism that generates, in HeLa cells, HDAC6 protein depletion in absence of Hsp27, we performed a quantification of *hdac6* gene product by quantitative PCR (qPCR). As shown in Fig. 3.E, the level of HDAC6 mRNA was not significantly altered in HSh27-2.2 cells compared to HMs27 cells. In order to determine if the level of HDAC6 protein was post-translationaly regulated, HeLa cells where treated with proteolysis inhibitors, such as the proteasome inhibitor MG132 or the calpain inhibitor ALLN. In our cells, MG132 was able to block the proteasomal pathway since the phenomenon correlated with poly-ubiquitin accumulation (Fig. 3.F). Both ALLN and MG132 up-regulated the level of HDAC6 in HSh27cells (Fig. 3G). Consequently, similar levels of HDAC6 were observed in every HeLa cells lines. This suggests that an increased degradation of HDAC6 occurs in the absence of Hsp27.

### Endogenous level of pro-caspase-3 is decreased in Hsp27 depleted cells

**Figure 4:**
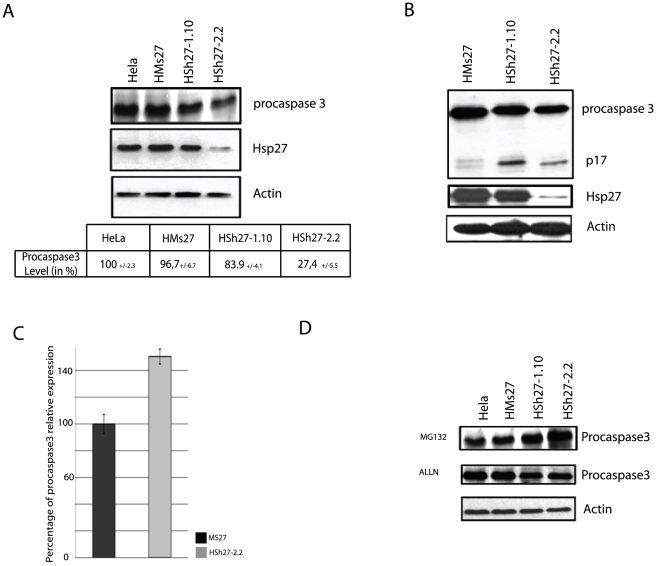
Knock out of Hsp27 induces procaspase-3 proteasomal degradation without modulating the expression of its gene. *A*, Total extracts of HeLa cell clones were analyzed in immunoblots incubated and revealed with caspase-3 antibody. Quantification of blots intensity was reported in a table. *B*, Each cell line was treated for 4 h with 0.25 µmol/L of staurosporine. Samples were analyzed in blot using anti caspase-3 antibody. *C*, Quantification of *procaspase-3* gene relative expression by qPCR experiments. *D*, Effect of proteolytic inhibitors. Samples were collected after being treated with MG132 or ALLN, as described in Fig. 3. Levels of procaspase-3 were revealed by western blot analysis.

Hsp27 has been described to interact with the pro-domain of procaspase-3, a key protease involved in the executive pathway of apoptosis [32]. Physical interaction with Hsp27 inhibits procaspase-3 processing leading to the activation of apoptotic caspases and participates to the mechanism of cell survival mediated by Hsp27 [7]. As shown in the immunoblots presented in Fig. 4 panel A, the level of endogenous procaspase-3 was down regulated in Hsp27 depleted HeLa clones. Quantification analysis revealed a 79.6% decrease in pro-caspase-3 level in the most Hsp27 depleted clone HSh27-2.2. As observed in transitory experiments, processing of procaspase-3 in p12 and p17 fragments after a 0.2 µmol/L staurosporine treatment was increased in all Hsp27 depleted HeLa clones (Figure 4B). In contrast, p12 and p17 cleaved fragments were not observed in the absence of apoptotic stimulation (data not shown) allowing us to conclude that the decrease in procaspase-3 level was not due to an increased procaspase-3 processing.

To investigate if the reduced level of procaspase-3 protein originated from an inhibition at the gene transcription level, *procaspase-3* gene product was quantified by qPCR (Fig. 4.C). Procaspase-3 mRNA was up regulated by 50% in HSh27-2.2 clone, indicating an increased in transcriptional activity when compared to control cells. As a consequence, these data strongly suggest a high rate of degradation of the protein. Thus, we tested procaspase-3 protein level when proteasomal and calpain pathways were inhibited by MG132 and ALLN, respectively (Fig. 4.D). Procaspase-3 level was drastically increased during MG132 treatment but was not restored by ALLN, suggesting an ubiquitin/proteasomal degradation in cells depleted of Hsp27. The analysis was not performed in MCF-7 cells because of the lack of expression of procaspase-3 in these cells.

### Hsp27 constitutive depletion induces STAT2 degradation and modulates STAT3 phosphorylation

**Figure 5:**
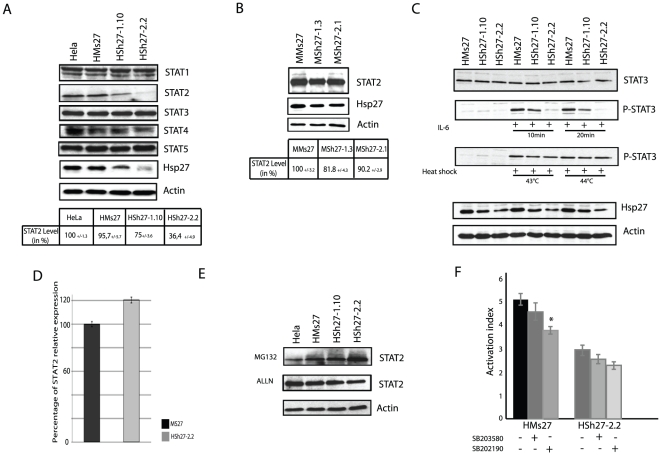
Endogenous level of STAT2 is decreased in Hsp27 depleted cell line. *A*, Hsp27 depleted and control HeLa cells were collected, and samples were analyzed by immunoblotting using antibodies that recognize the different STAT polypeptides. Quantification of STA2 level was reported in a table. *B*, as in *A*) but analysis of STAT2 level in MCF-7 depleted cells. C, Analysis of STAT3 phosphorylation. HeLa clones were treated with interleukin-6 (4 h; 125 ng/mL) or heat shock (60 min; 42°C and 44°C). Samples were collected and analyzed by immunobloting with STAT3, Phospho-STAT3, Hsp27 and actin antibodies. *D*, quantification of *stat2* gene relative expression by qPCR. *E*, Effect of proteolytic inhibitors. HeLa cells were analyzed after being treated with MG132 or ALLN, as described in Fig. 3. Level of STAT2 was revealed by immunobloting. *F*, HeLa cells were transiently transfected with an ISRE reporter gene construct [36] and treated for 6 h with 50 U/mL of interferon-α. They were also treated or not for 30 min with 10 µM of either SB203580 or SB202190 (p38MAPkinase and MK2 inhibitors, respectively) before exposure to interferon-α. Luciferase expression correlated with STAT2 transcriptional activity was quantified in both cell lines.

Hsp27 has been reported to interact with STAT3, a crucial transcription factor implicated in the maintenance and anti-apoptotic status of cancerous cells by activating the expression of genes like Bcl-XL and survivin [33], [34]. We then tested whether Hsp27 could interact with other member of the STAT family protein. As revealed by western blot analysis, the level of STAT2 was dramatically decreased in Hsp27 depleted HeLa clones (Figure 5.A). In contrast, other proteins of the STAT transcription factor family were not or only weakly (STAT4) modulated (Fig. 5.A). STAT2 transcription factor is implicated in processes such as viral or interferon responses and was not previously described to interact with Hsp27 [35]. Quantification of immunoblots performed with protein extracts from MCF-7 cell lines confirmed the results observed in HeLa cells, since STAT2 endogenous level decreased of 18.2% and 9,8% in the two MCF-7 partially depleted clones, respectively (Figure 5.B).

A preceding study has reported the depletion of STAT3 in prostate cells when Hsp27 was transitory decreased by antisense strategy [21]. In our conditions of constitutive depletion of Hsp27, endogenous level of STAT3 remained constant. STAT3 mRNA level was also not altered in Hsp27 depleted clones (Data S1.A). In contrast, STAT3 phosphorylation, induced by interleukin-6 (IL-6) or heat-shock at 43°C, was decreased in correlation to Hsp27 level. A confirmation of Rocchi *et al.* results was nevertheless obtained in transient transfection experiments of Sh27, suggesting a possible adaptation mechanism to the lack of Hsp27 in our constitutive clones (Data S1.B).

As proven by qPCR experiments, STAT2 messenger level was slightly increased in the characterized clones although the level of STAT2 polypeptide was reduced (Fig. 5.D). Furthermore, degradation of STAT2 was blocked when proteolytic degradation was inhibited by MG132 and ALLN treatments, implicating the crucial role of Hsp27 in the stability of this protein (Fig. 5.E).

STAT2 is a major transcription factor implicated in the interferon response [35]. To analyze its transcriptional activation in Hsp27 depleted cells, a DNA vector containing an ISRE element fused to luciferase reporter gene [36] was transfected in control and Hsp27 depleted HeLa cells. Cells were treated with interferon-α (50 U/ml) to activate the STAT2 interferon response. As seen in Fig. 5.F, luciferase expression was down regulated in HSh2.2 cells as compared to control cells, suggesting a decrease in STAT2 transcriptional activity. Moreover, we used two phosphorylation inhibitors, SB203580 and SB202190 (p38MAPkinase and MK2 inhibitors, respectively) to analyze the role of Hsp27 phosphorylation. Treatment with these inhibitors decreased STAT2 transcriptional activity (Fig. 5.F), hence suggesting a crucial role for Hsp27 and its phosphorylation in the regulation of STAT2 response to interferon-α.

### STAT2, HDAC6 and procaspase3 directly interact with Hsp27

**Figure 6:**
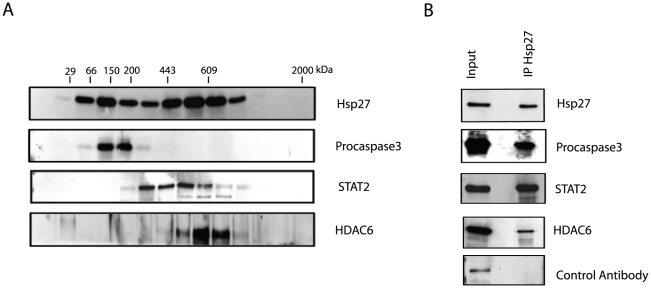
HeLa cells were lysed in the presence of 0.1% Triton X-100 and the 10,000×g soluble supernatants were applied to sepharose CL-6B gel filtration columns as described in Materials and methods. 29, 66, 150, 200, 443, 669 and 2000 (kDa) indicate the apparent native size of gel filtration markers. *A*, The presence of Hsp27, HDAC6, STAT2 and procaspase-3 in pooled fractions eluted from the columns was detected by western blot analysis. *B*, interactions between Hsp27 and client proteins analyzed by Co-IP experiments using a goat polyclonal anti-Hsp27 antibody. Column fractions corresponding to either procaspase-3, STAT2 or HDAC6 were pooled and used for immunoprecipitation. Immunoprecipitated proteins and input cell lysates were analyzed side by side in immunoblots probed with the indicated antibodies. A representative negative control of Co-IP was performed with a control antibody and revealed (here the anti procaspase 3).

In accordance with the “holdases” and “molecular sponges” theories [4], [37], we hypothesized that the chaperone activity of Hsp27 can stabilize polypeptides and modulates their half-life. The chaperone activity of Hsp27 is highly correlated with its structural organization. Indeed, this protein can form dynamic oligomeric structures up to 800 kDa [4], [38]. Hsp27 oligomerization profiles were then analyzed by the use of size exclusion columns (Fig. 6.A). In non-stressed cells, Hsp27 protein was currently present in two oligomeric populations with distinct native sizes, the small oligomers (≤200 kDa) and the large oligomeric structures over 200 kDa. Immunoblot analysis of the different eluted fractions revealed that procaspase-3 was only present in the fractions corresponding to Hsp27 population of small native sizes whereas STAT2 and HDAC6 proteins were only identified in higher, but distinct, molecular weights fractions (Fig. 6.A). Physical interactions between Hsp27 and procaspase3, HDAC6 and STAT2 were tested by Co-IP experiments on column fractions. Goat polyclonal antibody raised against Hsp27 was added to pooled column fractions that were positive for the targetted proteins (native sizes of about 100–200 kDa in the case of procaspase-3, 250–500 kDa in the case of STAT2 and 550–650 kDa in the case of HDAC6). Immunoblots were revealed with the corresponding antibody (Fig. 6.B). These results demonstrate a strong interaction between the three tested polypeptides and Hsp27. Interactions were restrained to sub-fractions of total Hsp27 that are characterized by a specific level of oligomerization. Hence, these interactions should highly correlate with specific structural organizations of the different partners. However, we cannot exclude that the medium and large populations could also include interacting complex formed by HspB1 small oligomers with several protein partners.

## Discussion

In the present study, we characterized cancerous HeLa and MCF-7 human cells lines that show reduced level of Hsp27 expression consequently of the transient or constitutive expression of shRNA targeting Hsp27 mRNA. Isolation of Hsp27 depleted stable clones was a rather difficult task since few cells survived the selection, a phenomenon that could be linked to the lack of Hsp27 mediated protective activity. We noticed in the survival clones that the constitutive knock down or reduced levels of Hsp27 did not modulate the levels of major Hsps, as Hsp70 and 90. In contrast, pro-apoptotic proteins like Bax and Bid were over-expressed in both HeLa and MCF-7 cells, a possible response towards the increased tendency of cancer cells under-expressing Hsp27 to undergo spontaneous apoptosis [4], [8], [17], [21]. On the other hand, over-expression of survival proteins like survivin (HeLa cells) or Bcl-2 (MCF-7 cells) could result of adaptation mechanisms to survive in the presence of low levels or absence of Hsp27.

Of interest, the constitutive knock down of Hsp27 induced the degradation of HDAC6, STAT2 and procaspase-3 proteins without down regulating their mRNA transcripts levels. Furthermore, we demonstrated a biochemical interaction between different oligomeric forms of Hsp27 and these specific polypeptides. This highly suggests a new molecular chaperoning function of Hsp27 towards native polypeptides.

Additionally, the slow growing HeLa clones, able to adapt to Hsp27 knock-down, displayed nucleus abnormalities and polyploidy. The alteration in cytokinesis efficiency was probably the origin of the increased number of cells in G2/M phase. This may be related to the activation of cell senescence, as observed by others [27]. In HeLa cells, part of the nuclear phenotype associated with Hsp27 depletion could have resulted of α-tubulin hyper-acetylation consequently to HDAC6 degradation. In that regard it is interesting to note that HDAC6 is a major component of the cellular stress response, together with the major heat shock proteins [39], which participates in the addressing of denatured proteins to degradation [40].

In response to heat shock, large oligomers of Hsp27 store unfolded and denatured proteins, particularly when the molecular chaperone pathway is saturated [41]. In the altered protein response of cells, the first link between Hsp27 and HDAC6 is their direct interaction. Secondly, HDAC6 is able to promote *hsp27* gene transcription linked to stress response [42]. Furthermore, it has been reported that HDAC6 is a powerful modifier of carcinogenesis and its stabilization by Hsp27 may contribute to oncogenic pathways activation [43].

Procaspase-3 is a major cysteine protease involved in apoptosis and differentiation pathways [44], [45]. An interaction has already been described between the pro-domain of procaspase-3 and Hsp27, which modulates procaspase-3 cleavage and activation [32]. Our new finding implicates that, in HeLa cells, Hsp27 could regulate procaspase-3 half-life. We were not able to realize the same experiments in MCF-7 breast cancer cells because caspase-3 is inactivated due to a deletion mutation in exon 3 of the gene [46]. Our results are in accordance with a previous study that has already described a procaspase-3 down-regulation when Hsp27 is immuno-depleted [7]. However, the authors suggested that the down-regulation of procaspase-3 was a consequence of its cleavage in functionally caspase-3. In contrast, our results suggest that, in the absence of Hsp27, procaspase-3 is rapidly degraded through the ubiquitin/proteasome pathway.

STAT2 is a central transcription factor implicated in interferon and anti-viral responses [35]. We show here that STAT2, which interacts with Hsp27 large oligomers, was degraded in Hsp27 depleted stable cells. In these cell lines, we were unable to detect a decrease in the level of STAT3, a protein that has already been demonstrated to interact with Hsp27 [21], [33]. However, we found that the phosphorylation of STAT3, which reflects its transcriptional activation, was down-modulated in response to IL-6 and also heat shock. However, in cells transiently transfected with shRNA encoding vector, we confirmed that STAT3 was degraded and its phosphorylation down-regulated. Hence, in our cell conditions, the establishment of stable cell lines altered the degradation of STAT3 in absence of Hsp27. This reflects an adaptation mechanism as well as the tight regulation that controls STAT3 stabilization and activation [21].

It can be hypothesized that, in cancer cells, the abundance of Hsp27 may, similarly to Hsp90 [20], contribute to the stabilization of several oncogenic proteins. One example of such an activity refers to the function of Hsp27 in bortezomib treated cells where the proto-oncogene STAT3 is constitutively activated [15], [21], [47]. Another example, in the context of apoptosis after androgen ablation and chemotherapy, concerns the translation initiation factor 4E (eIF4E) that shows a decreased expression at the protein, but not mRNA, level in cells depleted of Hsp27 [22]. Interaction with eIF4E was confirmed but the active oligomeric size of Hsp27 is still not known. Based on the work dealing with Hsp90, a protein which, in yeast, appears to stabilize approximately 10% of the proteome [18], the number of client proteins interacting with Hsp27 complex oligomeric and phosphorylated sub-structures [4] could also be very high. Indeed, Hsp27 has been referred to act as “holdase” or “molecular sponge”. Nevertheless, identification of Hsp27 client proteins is a difficult task. For example, a powerful mass-spectrometry study identified an important number of proteins that bind sHsps after heat shock [48], but this approach was not successful in non-stressed conditions.

The results presented here suggest that at least three new polypeptides: HDAC6, STAT2 and procaspase-3 could be client proteins of Hsp27. Hence, it becomes more and more evident that, in unstressed cells, Hsp27 acts as a key regulator of a pleiotropic number of target proteins. Based on the Hsp90 chaperoning model, our findings support the hypothesis that Hsp27 has its own client proteins; a property that may be shared by Hsp chaperones. However, whether Hsp27, similarly to Hsp90, could stabilize mutant forms of specific proteins is not yet known. The results presented here also suggest that, in cancer cells, rational design of stable peptides, based on the Shepherdin model [49], or chemical drugs could be an approach to target Hsp27 oncogenic functions by counteracting its interaction with specific oncogenic client proteins.

## Supporting Information

Data S1 *A*, quantification of *stat3* gene relative expression by qPCR analysis. *B*, HeLa cells were transitively transfected with Sh27 or Ms27. 48 h after transfection, samples were collected and analyzed by western blot with STAT3 antibody.
(EPS)
